# Spontaneous Involution of a Presumably Rathke's Cleft Cyst in a Patient with Slight Subclinical Hypopituitarism: A Case Report and Review of the Literature

**DOI:** 10.1155/2015/971364

**Published:** 2015-08-27

**Authors:** Diaa Al Safatli, Rolf Kalff, Albrecht Waschke

**Affiliations:** Department of Neurosurgery, Friedrich-Schiller University of Jena, Erlanger Allee 101, 07747 Jena, Germany

## Abstract

Rathke cleft cyst is described as benign intrasellar cyst. They are mostly small and asymptomatic; they may become large enough to cause symptoms by compression of intrasellar or suprasellar structures. We report on a case of spontaneous regression of a symptomatic RCC with subsequent recovery of preexisting endocrine dysfunction and resolution of headaches. A 60-year-old man complained about headaches. Laboratory investigation revealed a partial hypopituitarism with a slight central hypothyroidism without need for substitution. An MRI study showed a cystic, T2-hyperintense, sellar lesion compatible with a RCC. At one year follow-up, the patient had no complaints and the hormone work-up revealed a regression of the previous slight hypopituitarism. The MRI study showed a complete regression of the cystic lesion and a normal sized and shaped pituitary gland. The spontaneous regression of cystic sellar lesions is rare. The exact mechanism of the possible spontaneous involution of RCC is until now not well understood. However, spontaneous regression is possible and justifies the conservative therapy with regular clinical and radiological follow-up for asymptomatic patients or patients with symptoms not caused by the mass effect of these lesions.

## 1. Introduction

Rathke's cleft cyst (RCC) is first described by Luschka in 1860. RCC is a benign cystic lesion in the sella turcica region that arises from the residual of Rathke's pouch [1]. They are lined with single layer of cuboidal epithelium and containing mucoid material [2]. These cysts are most frequently small and asymptomatic; they may become occasionally large enough to cause symptoms by compression of intrasellar or suprasellar structures [3, 4].

Although asymptomatic patients undergo conservative treatment, patients with symptoms are typically treated surgically [3]. Rare cases of spontaneous involution of cystic sellar lesions are to be found in the medical literature. The mechanism of this possible regression remains poorly understood [5–7].

In the current report, we present the case of a cystic sellar lesion, most likely RCC, in a 60-year-old man, with slight hypopituitarism. The patient showed one year after the initial diagnosis a spontaneous complete radiological regression of this cystic sellar lesion, as well a normal pituitary gland function. We discuss the case and review the literature.

## 2. Case Report

A 60-year-old man complained about headache after a plastic surgery of the tympanic membrane at another hospital; there was no weakness and no visual symptoms. His medical history was otherwise unremarkable. The initial general physical examination in our outpatient clinic was unremarkable. The neurological examination showed an awake, alert, completely orientated patient with no focal signs. The visual field as well the formal ophthalmologic examination was within the normal limits. A hormone profile revealed a slight central hypothyroidism with no need for hormone substitution therapy. An MRI study revealed a cystic lesion in the sella turcica region with a thin contrast-enhancing rim on the left peripheral side of the lesion. The lesion was isotense compared to brain on T1-weighted MRT and hyperintense on T2-weighted imaging, measuring 17 × 15 × 12 mm. There was no intracystic nodule. There was no displacement or compression of the optic apparatus. A computed tomographic scan (CT) demonstrated a slightly enlarged sella turcica but no abnormal calcifications. Because of the radiological findings, the differential diagnosis included firstly Rathke's cleft cyst (RCC) und less likely a craniopharyngioma or cystic pituitary adenoma.

The management options for this lesion including the surgical und the conservative options were discussed in detail with the patient. Given that the clinical, neurological, and ophthalmological examination was within normal limits and given the unlikely regression of the slight hypopituitarism after surgery, we saw no urgent indication for surgical treatment and choose the close clinical, ophthalmological, and radiological observation.

At the 3-month follow-up evaluation, the patient reported a complete regression of the headache and no other complaints. The MRT study showed no remarkable change in size and form of the known lesion and still no displacement of the optic apparatus.

At the 6-month follow-up, there were no clinical complaints. The neurological and ophthalmological examination was also completely normal. The MRI study continued to show no remarkable difference compared to previous studies.

**Figure 1:**
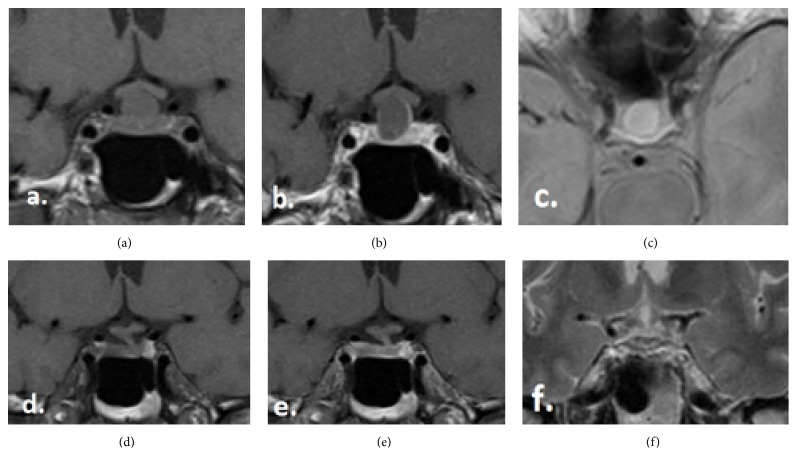
(a–c) The MRI images by the initial diagnosis, (a) T1-coronal without contrast, (b) T1-coronal with contrast, and (c) T2-axial without contrast. (d–f) The MRI images by the 1-year follow-up, (d) T1-coronal without contrast, (e) T1-coronal with contrast, and (f) T2-coronal without contrast.

At 1-year follow-up evaluation, the patient had no complaints. The neurological status, as well the visual field, continued to be normal. The hormone work-up revealed regression of the previous slight hypopituitarism showing normal hormone profile. The MRT study demonstrates a complete involution of the cystic lesion, most likely RCC, in the sellar region and showed a normal size and shape of the pituitary gland with no contrast enhancement (Figure 1).

At the time, when this paper was written (24 months after the initial diagnosis), the patient has still no complaints and no signs of relapse on MRI. In the meantime, the patient did not undergo any surgery or medical or emotional stress, so that no steroid stress injections became necessary.

## 3. Discussion

Rathke cleft cysts are classically described as benign epithelium-lined intrasellar cysts containing mucoid material. The MRI appearance of RCC is highly variable. However, a few key findings that may help to distinguish RCC from other cystic lesions in the sella turcica region have been reported [8]. RCCs are typically well-defined, round, noncalcified lesions within or adjacent to the pituitary gland, which usually do not exhibit destruction or enlargement of the sella turcica [9]. RCCs are almost always homogeneous in MR intensity, except for waxy nodules. Nishioka et al. studied 27 patients with histologically proven RCC and 10 patients with a radiological diagnosis of RCC and suggested that the presence of waxy nodules, though not found in every case, is the most reliable diagnostic indicator of RCCs [10]. CT density and MRI intensity of RCCs vary considerably depending on the cystic content. A high T1-WI intensity has been interpreted to indicate a high content of protein and mucopolysaccharide and rarely hemorrhage. Calcification is commonly seen in craniopharyngiomas, but very rarely in other cystic lesions [11–15]. The patient in our present case exhibited a cystic lesion in the sella turcica lesion, which was isotense compared to brain on T1-weighted MRT and hyperintense on T2-weighted imaging, suggesting mucus with low viscosity. There was in our case no intracystic nodule and a very thin enhancing rim. A computed tomographic (CT) scan demonstrated no abnormal calcifications. According to this neuroimaging aspect, we regarded the cyst described in our report very likely to be a benign Rathke's cleft cyst.

Cyst size does not correlate with the presence of pituitary dysfunction. This is different from the situation with adenomas, which usually develop hypopituitarism only when they become large tumours. Saeki et al. reported that RCCs with isointensity to high intensity on T1-WI cause clinical symptoms with smaller size than cysts of low intensity; thus, it is considered that chronic inflammation participates in the development of some cases of pituitary dysfunction [10, 16]. In our case, the lesion was isotense on T1 and measured 17 × 15 × 12 mm; thus, we think a combination of possible inflammation and direct mass effect is responsible for the slight hypopituitarism.

The pituitary hemorrhage usually occurs into a pituitary adenoma; hemorrhage into other sellar lesions including a Rathke's cleft cyst has also been reported [12–14]. Therefore, the pituitary apoplexy has to be considered in the differential diagnosis. The headache in our case was not severe and with no sudden onset, which is typical for such hemorrhage und there was no apparent evidence of hemorrhage on the neuroimaging studies. For this reason, we considered the pituitary apoplexy for unlikely diagnosis.

The spontaneous regression of cystic sellar lesions is rare. We found in our literature search 10 other published articles (Table 1). J. D. Simmons and L. A. Simmons presented a pituitary cyst discovered on MRI in an amenorrheic patient that regressed over 3 months [15]. Igarashi et al. reported four asymptomatic cystic sellar tumors that shrank or completely disappeared spontaneously [13]. Saeki et al. reported two similar cases in their published study about fluctuating visual field defects in Rathke's cleft cysts [17]. Terao et al. described a case of a suprasellar cystic tumor by a 67-year-old man complaining of bitemporal homonymous hemianopsia. The MRI taken 5 years after the initial MRI revealed a spontaneous disappearance of the lesion [18]. Nishio et al. published two cases of pituitary cysts, which regressed over months. They assumed that this cystic lesion shrank after “pituitary apoplexy” [14]. Nishioka et al. reported also two cases with regression of the size of RCC in their study about magnetic resonance imaging, clinical manifestations, and management of Rathke's cleft cyst [10]. Maruyama et al. described a RCC case by an 81-year-old man with panhypopituitarism and central diabetes insipidus. The cystic mass shrank following the start of glucocorticoid replacement. However, because of the concern over possible side effects of supraphysiological doses of glucocorticoid replacement, surgical treatment was carried out, confirming the histopathological feature of Rathke's cleft cyst [19]. Amhaz et al. reported 9 patients with cystic sellar lesions and imaging characteristics consistent with an RCC; in all cases, there was spontaneous involution of the lesions [3]. Maniec and Watson presented a case of spontaneous rupture and complete disappearance of a sella-suprasellar cyst followed by a remarkable rapid cyst reaccumulation requiring surgery, providing an evidence that an “empty sella syndrome” may indeed need clinical follow-up [5]. Munich and Leonardo described a case of RCC in an 8.5-year-old girl who presented with a history of growth deceleration since 4 years of age. Endocrine evaluation revealed growth hormone deficiency, central hypothyroidism, and diabetes insipidus, but normal cortisol secretion. The patient was treated medically for her hormone deficiencies. Over the next year, her sellar mass involuted spontaneously [7]. However, to our knowledge, this is the first case Report with spontaneous regression of the partial hypopituitarism simultaneously with the radiological Involution of the cystic sellar lesion without need for hormone replacement therapy.

The mechanism of regression is not well understood. Some authors suggested that the changes in cyst size are due to imbalances between secretion and absorption of cystic fluid [3, 13, 15]; others suggested that repeated cyst ruptures might be the main factor [3, 14]. Our case lacks the histological confirmation of an RCC, but radiological features were consistent with this diagnosis of RCC and exclude with reasonable surety the other cystic lesions of the sella.

## 4. Conclusion

The exact mechanism of the possible spontaneous involution of RCC is until now not well understood, as well as the course of development of these lesion, including the possibility of reaccumulation after a previous regression. However, the spontaneous regression is possible and justifies the conservative therapy with regular clinical and radiological follow-up for asymptomatic patients or patients with symptoms not caused by the mass effect of these lesions.

## Figures and Tables

**Table 1:** Reported cases of spontaneous regression of RCC.

Case number	Authors and year	Age (yrs), sex	Symptoms	Time to regression (mos)	Endocrine evaluation
1	J. D. Simmons and L. A. Simmons, 1999 [15]	15, F	Amenorrhea	3	Low LH, FSH, and estradiol

2	Igarashi et al., 1999 [13]	25, F	Visual field defect	NA	NA
		46, M	Visual field defect	NA	NA
		34, M	Visual field defect	NA	NA
		58, F	Visual field defect	NA	NA

3	Saeki et al., 1999 [17]	59, F	Visual field defect	1	Normal
		30, M	Visual field defect	0.5	Low LH, FSH, and GH

4	Terao et al., 2001 [18]	67, M	Visual field defect	60	NA

5	Nishio et al., 2001 [14]	14, M	Headache	3	Normal
		31, F	Headache	6	Normal

6	Nishioka et al., 2006 [10]	NA	NA	NA	Hypopituitarism
		NA	Visual field defect	NA	NA

7	Maruyama et al., 2008 [19]	81, M	Headache	0.5	Hypopituitarism

8	Amhaz et al., 2010 [3]	57, M	Headache	5	Low testosterone
		32, F	Headache	44	Normal
		29, F	None	31	Normal
		18, F	Headache	7	Elevated ACTH
		17, F	Headache	21	Normal
		14, M	Headache	11	GH slightly high
		6, M	Headache	41	Normal
		5, M	Growth	100	Low testosterone and TSH
		29, F	deceleration Headache	18	Normal

9	Maniec and Watson, 2011 [5]	59, M	Headache	6	NA

10	Munich and Leonardo, 2012 [7]	8.5, F	Growth deceleration	12	Low GH, central hypothyroidism

NA, information not available in paper; M, male; F, Female; mons, months; yrs, years; ACTH, adrenocorticotropic hormone; TSH, thyroid-stimulating hormone; T3, triiodothyronine; T4, thyroxine; GH, growth hormone; LH, luteinizing hormone; FSH, follicle-stimulation hormone.

## References

[1] Noh S. J., Ahn J. Y., Lee K. S., Kim S. H. (2007). Pituitary adenoma and concomitant Rathke's cleft cyst. *Acta Neurochirurgica*.

[2] Voelker J. L., Campbell R. L., Muller J. (1991). Clinical, radiographic, and pathological features of symptomatic Rathke's cleft cysts. *Journal of Neurosurgery*.

[3] Amhaz H. H., Chamoun R. B., Waguespack S. G., Shah K., McCutcheon I. E. (2010). Spontaneous involution of Rathke cleft cysts: is it rare or just underreported?. *Journal of Neurosurgery*.

[4] Zada G. (2011). Rathke cleft cysts: a review of clinical and surgical management. *Neurosurgical Focus*.

[5] Maniec K., Watson J. C. (2011). Spontaneous rupture, disappearance, and reaccumulation of a Rathke's cleft cyst. *Case Reports in Endocrinology*.

[6] Sade B., Albrecht S., Assimakopoulos P., Vézina J.-L., Mohr G. (2005). Management of Rathke's cleft cysts. *Surgical Neurology*.

[7] Munich S. A., Leonardo J. (2012). Spontaneous involution of a Rathke's cleft cyst in a patient with normal cortisol secretion. *Surgical Neurology International*.

[8] Steinberg G. K., Koenig G. H., Golden J. B. (1982). Symptomatic Rathke's cleft cysts. Report of two cases. *Journal of Neurosurgery*.

[9] Oka H., Kawano N., Suwa T. (1994). Radiological study of symptomatic Rathke's cleft cysts. *Neurosurgery*.

[10] Nishioka H., Haraoka J., Izawa H., Ikeda Y. (2006). Magnetic resonance imaging, clinical manifestations, and management of Rathke's cleft cyst. *Clinical Endocrinology*.

[11] Lee C.-H., Seo E.-K., Cho Y.-J., Kim S.-J. (2008). Large ossified Rathke's cleft cyst—a case report and review of the literature. *Journal of Korean Neurosurgical Society*.

[12] Donovan L. E., Corenblum B. (1995). The natural history of the pituitary incidentaloma. *Archives of Internal Medicine*.

[13] Igarashi T., Saeki N., Yamaura A. (1999). Long-term magnetic resonance imaging follow-up of asymptomatic sellar tumors—their natural history and surgical indication. *Neurologia Medico-Chirurgica*.

[14] Nishio S., Morioka T., Suzuki S., Fukui M. (2001). Spontaneous regression of a pituitary cyst: report of two cases. *Clinical Imaging*.

[15] Simmons J. D., Simmons L. A. (1999). Spontaneous regression of a pituitary cyst. *Neuroradiology*.

[16] Saeki N., Sunami K., Sugaya Y., Yamaura A. (1999). MRI findings and clinical manifestations in Rathke's cleft cyst. *Acta Neurochirurgica*.

[17] Saeki N., Kubota M., Yamaura A., Ishige N. (1999). Fluctuating visual field defects in Rathke's cleft cysts: MRI analysis. *Journal of Clinical Neuroscience*.

[18] Terao T., Sawauchi S., Hashimoto T., Miyazaki Y., Akiba Y., Abe T. (2001). A case of spontaneous rupture of a suprasellar cystic mass. *No Shinkei Geka*.

[19] Maruyama H., Iwasaki Y., Tsugita M. (2008). Rathke's cleft cyst with short-term size changes in response to glucocorticoid replacement. *Endocrine Journal*.

